# Active fraction (HS7) from *Taiwanofungus camphoratus* inhibits AKT-mTOR, ERK and STAT3 pathways and induces CDK inhibitors in CL1-0 human lung cancer cells

**DOI:** 10.1186/s13020-017-0154-9

**Published:** 2017-11-15

**Authors:** I-Chun Lai, Gi-Ming Lai, Jyh-Ming Chow, Hsin-Lun Lee, Chuan-Feng Yeh, Chi-Han Li, Jiann-Long Yan, Shuang-En Chuang, Jacqueline Whang-Peng, Kuan-Jen Bai, Chih-Jung Yao

**Affiliations:** 1 Division of Radiation Oncology, Department of Oncology, Taipei Veterans General Hospital, Taipei, 11217 Taiwan; 2 Cancer Center, Wan Fang Hospital, Taipei Medical University, Taipei, 11696 Taiwan; 3 Comprehensive Cancer Center of Taipei Medical University, Taipei, 11031 Taiwan; 4 Department of Internal Medicine, School of Medicine, College of Medicine, Taipei Medical University, Taipei, 11031 Taiwan; 5 Division of Hematology and Medical Oncology, Wan Fang Hospital, Taipei Medical University, Taipei, 11696 Taiwan; 6 National Institute of Cancer Research, National Health Research Institutes, Miaoli County, 35053 Taiwan; 7 Department of Radiation Oncology, Taipei Medical University Hospital, Taipei Medical University, Taipei, 11031 Taiwan; 8 Division of Pulmonary Medicine, Department of Internal Medicine, Wan Fang Hospital, Taipei Medical University, Taipei, 11696 Taiwan; 9 School of Respiratory Therapy, College of Medicine, Taipei Medical University, Taipei, 11031 Taiwan; 10 Wan Fang Hospital, Taipei Medical University, Taipei, 11696 Taiwan

**Keywords:** Non-small cell lung cancer, HS7, *Taiwanofungus camphoratus*, AKT-mTOR, ERK, STAT3

## Abstract

**Background:**

The non-small cell lung cancer (NSCLC) is the leading cause of cancer death worldwide. In NSCLC, the oncogenic AKT-mTOR, ERK and STAT3 pathways are commonly dysregulated and have emerged as attractive targets for therapeutic developments. In a relatively limited subset of NSCLC, these pathways driven by mutant EGFR can be treated by the tyrosine kinase inhibitors (TKIs)-mediated targeted therapy. However, for the most NSCLC, more novel targeted agents are imperatively needed. Therefore, we investigated the inhibitory effects of the active fraction HS7 from *Taiwanofungus camphoratus*, a unique medicinal fungus in Taiwan, on these pathways in CL1-0 EGFR wild-type human NSCLC cells.

**Methods:**

The active fraction HS7 was prepared by *n*-hexane extraction of *T. camphoratus* followed by silica gel chromatography. Its effects on the cell viabilities were determined by sulforhodamine B colorimetric assay. Flow cytometry was used to analyze cell-cycle regulation and apoptosis induction. The changes in cellular protein levels were examined by Western blot.

**Results:**

The active fraction HS7 vigorously inhibits AKT-mTOR, ERK and STAT3 signaling pathways in CL1-0 cells. At dose of 25 μg/mL, these signaling pathways were almost completely inhibited by HS7, accompanied with induction of cyclin-dependent kinase inhibitors such as p15, p21 and p27. Accordingly, the AKT-mTOR downstream targets p-p70S6K and HIF-1α were also suppressed as well. At this dose, the cell proliferation was profoundly suppressed to 23.4% of control and apoptosis induction was observed.

**Conclusions:**

The active fraction HS7 from *n*-hexane extract of *T. camphoratus* exerts multi-targeting activity on the suppression of AKT-mTOR, ERK and STAT3 pathways and induction of p15, p21 and p27 in EGFR wild-type NSCLC cells. This multi-targeting activity of HS7 suggests its potential as an alternative medicine for the treatment of EGFR TKIs resistant NSCLC.

**Electronic supplementary material:**

The online version of this article (10.1186/s13020-017-0154-9) contains supplementary material, which is available to authorized users.

## Background

Lung cancer is the leading cause of cancer death worldwide and its incidence is still on the increase [1]. About 89% of lung cancer patients are non-small cell lung cancer (NSCLC) [2] and 20–30% of these patients were diagnosed at a locally advanced stage [3]. In most NSCLC patients, the oncogenic AKT, ERK and STAT3 signaling pathways are constitutively activated. It has been reported that 50–70% overexpression of phosphorylated AKT [4], 70% expression of activated ERK [5] and over 50% high levels of activated STAT3 [6] were observed in NSCLC. Aberrant activation of these three signaling pathways results in uncontrolled proliferation, apoptosis resistance and other oncogenic cascades in lung cancer cells [4, 6–8]. Therefore, there has been increasing research interest in identifying novel therapeutics to target these oncogenic signaling pathways for effectively treating NSCLC patients [4, 9, 10].

PI3K-AKT-mTOR and RAS-RAF-MEK-ERK are two main downstream signaling pathways driven by *epidermal growth factor receptor* (*EGFR*) mutations or abnormal fusion of *echinoderm microtubule*-*associated protein*-*like 4* and *anaplastic lymphoma kinase* (*EML4*-*ALK*) genes [4]. STAT3 (signal transducer and activator of transcription 3), an important point of convergence for various signaling pathways [6] is also required for the oncogenic effects of NSCLC-associated *EGFR* mutations [11]. The relatively limited subset of NSCLC carrying the above genetic mutations can be effectively treated by the tyrosine kinase inhibitors (TKIs)-mediated targeted therapy [4, 12, 13]. However, most NSCLC patients do not harbor these genomic events and the 5-year survival rate remains dismal [13]. More novel targeted agents to suppress these oncogenic pathways are imperatively needed.

*Taiwanofungus camphoratus* (syn. *Antrodia camphorata*) is a widely used medicinal polypore fungi, which belongs to the Polyporaceae, Basidiomycotine family, and grows in a unique host, the endemic perennial tree *Cinnamomun kanehirai* (Bull camphor tree) in Taiwan [14, 15]. It is a well-known folk medicine and has been used as a local remedy to treat abdominal pain, diarrhea, drug intoxication, hypertension, and skin itching as well as to improve immune system and liver function [15, 16]. On the other hand, many studies have demonstrated its anticancer effects in the aspects of anti-proliferation, apoptosis induction and anti-invasion [14, 16–18]. Instead of the commonly used ethanol, we use *n*-hexane to extract the *T. camphoratus* and further separate the extract into eight fractions (HS1–HS8) by silica gel chromatography. We have isolated the most potent active fraction (HS7) according to its anti-proliferative activities against a panel of human cancer cell lines, including, lung (CL1-0), prostate cancer (PC3) and hepatocellular carcinoma (HepG2, Hep3B and Huh7) cells (see Additional file 1). Our previous study had demonstrated the effects of HS7 on the apoptosis induction and Wnt/β-catenin signaling inhibition in human colon cancer cells [14]. In the present study, we explore its effects on the aforementioned AKT-mTOR, ERK and STAT3 signaling pathways in a human NSCLC cell line CL1-0, which harbors wild-type EGFR and is resistant to EGFR TKIs [19]. The results show that HS7 vigorously suppresses the signaling pathways described above and arrests the cell growth, accompanied with induction of cyclin-dependent kinase (CDK) inhibitors such as p15, p21 and p27. Our findings suggest the potential of HS7 as an alternative medicine for the treatment of NSCLC.

## Methods

### Information of experimental design and resources

The information of experimental design, statistics, and resources used in this study are attached in Minimum standards of reporting checklist (Additional file 2).

### Cell culture

The CL1-0 human lung adenocarcinoma cell line was kindly provided by Dr. Shine-Gwo Shiah (NHRI, Miaoli, Taiwan) and the MRC-5 normal fetal human lung fibroblasts were purchased from Bioresource Collection and Research Center (Hsinchu, Taiwan). CL1-0 cells were maintained in RPMI1640 (Gibco, CA, USA) and MRC-5 cells were in MEM (Gibco, CA, USA) medium supplemented with 10% fetal bovine serum and 1× penicillin–streptomycin–glutamine (Gibco, CA, USA). Cells were cultured at 37 °C in a water-jacketed 5% CO_2_ incubator.

### Preparation of ethanol and *n*-hexane extracts of *T. camphoratus* and the active fraction HS7

As shown in our previous study [14], the fruiting body-like *T. camphoratus* (Voucher Number TC-2004-09-001) was cultivated and provided by Well Shine Biotechnology Development Co. (Taipei, Taiwan). Briefly, air-dried ground powder of cultivated *T. camphoratus* was extracted exhaustively with *n*-hexane or ethanol. The *n*-hexane extract was then further separated to eight fractions (HS1–HS8) by silica gel chromatography, and the seventh fraction (HS7) exerted the most potent effect on the growth inhibition of a screening panel of cancer cell lines (CL1-0, PC3, HepG2, Hep3B and Huh7). After lyophilization, the stock solutions of ethanol and *n*-hexane extracts and HS7 dissolved in dimethyl sulfoxide (DMSO) at concentration of 100 mg/mL were made. They were diluted in phosphate-buffered saline (PBS) prior to use. The final concentrations of DMSO were all below 0.2%.

### Reagents

The sulforhodamine B (SRB) dye for cell viability assay, propidium iodide for cell cycle analysis and the EGFR-TKI gefitinib (Iressa) were from Sigma-Aldrich (St. Louis, MO, USA). The MEK-ERK pathway inhibitor U0126 and PI3K-AKT pathway inhibitor LY294002 were from Cell Signaling Technology (Danvers, MA, USA). The JAK-STAT3 pathway inhibitor AG490 was from Calbiochem (La Jolla, CA, USA).

### Cell viability assay by sulforhodamine B (SRB) staining

According to the method described by Vichai and Kirtikara [20], SRB dye-binding assay was used to determine the viability of cancer cells. CL1-0 and MRC-5 cells were seeded in a 96-well plate at a density of 2 × 10^3^ cells/well in 10% FBS-RPMI or MEM medium. After 24 h of incubation, cells were treated with agents as indicated or PBS only for another 72 h. Cells were then harvested and fixed by 10% trichloroacetic acid (TCA). After fixing, cells were washed by distilled water and stained viable cells by 0.4% (w/v) SRB dye dissolved in 1% acetic acid. After staining for 30 min, the unbound dye was then washed away by 1% acetic acid and the plate was air-dried. The cell-bound SRB dye was then dissolved in 200 μL of 10 mM Tris base and the absorbance was read on a microplate reader (Molecular Devices, CA, USA) at a wavelength of 562 nm. The absorbance was directly proportional to the cell number over a wide range.

### Cell cycle analysis

Propidium iodide (PI) staining and flow cytometry were used to determine the cell cycle distribution. One day after being seeded in a six-well plate (10^5^ cells/2 mL/well), CL1-0 cells were treated with different doses of HS7 for 72 h. At harvest, cells were washed with PBS, incubated with 0.25% Trypsin EDTA at 37 °C for 5–10 min and then suspended in medium at a concentration of 1 × 10^6^ cells/tube. After being washed with PBS and centrifuged at 1200 rpm at 4 °C for 5 min, cells were resuspended in 500 μL PBS and fixed with 70% ethanol followed by gentle vortexing. Cells were allowed to stand overnight at − 20 °C. Fixed cells were spun down and washed with PBS. The cells were suspended in 500 μL PI (2 μg/mL)/Triton X-100 (0.1% v/v) staining solution with RNase A (200 μg/mL) at room temperature for 20 min and then analyzed by a flow cytometer (FACSCalibur™, BD Bioscience, CA, USA). Approximately 10,000 counts were made for each sample. The percentages of cell-cycle distribution were calculated by CellQuest software (BD Bioscience, CA, USA).

### Western blotting

A total of 4 × 10^5^ CL1-0 cells/10 cm dish were incubated for 24 h after seeding and then treated as indicated in figures for 72 h. On the day of harvest, the whole-cell lysates were prepared with radioimmunoprecipitation (RIPA) lysis buffer containing 1× tyrosine phosphatase inhibitor cocktail (FC0020-0001, BIONOVAS, Toronto, Canada), 1× protease inhibitor cocktail-full range (FC0070-0001, BIONOVAS, Toronto, Canada), and 1× serine/threonine phosphatase inhibitor cocktail (FC0030-0001, BIONOVAS, Toronto, Canada). Samples of protein extract were size fractionated electrophoretically by polyacrylamide SDS-PAGE gel and transferred onto a PVDF membrane using the BioRad Mini Protean electrotransfer system (CA, USA). The membranes blots were incubated with 5% milk in PBST for 1 h to block nonspecific binding and then were incubated with primary antibodies overnight at 4 °C. The membranes were detected with an appropriate peroxidase-conjugated secondary antibody incubated at room temperature for 1 h. Intensive PBS washing was performed after each time of incubation. The immune complexes were visualized using an enhanced chemiluminescence detection system (ECL, Perkin Elmer, Waltham, MA, USA) according to the manufacturer’s instructions. Primary antibodies against ERK (sc-94), p-ERK (sc-7383), STAT3 (sc-8019), p-STAT3 (sc-7993), p-p70S6K (sc-7984-R), p15 (sc-613), tubulin (sc-5286) and GAPDH (sc-47724) were purchased from Santa Cruz Biotechnology (San Diego, CA, USA). Primary antibodies for p-AKT (#4051), p-mTOR (#2971), mTOR (#2972) and p-pRB (#9308) were purchased from Cell Signaling Technology (Danvers, MA, USA). Primary antibodies for p21 (#05-345) and HIF-1α (07-628) were purchased from Upstate Biotechnology (Lake Placid, NY, USA). Primary antibody for p27 (#610241) was purchased from BD Transduction Laboratories (San Jose, CA, USA).

### Photograph of the cells

At harvest, cells were examined under a Nikon inverted microscope. The photomicrographs were captured by a CCD camera (Nikon, Mito, Japan) adapted to the microscope.

### Statistical analysis

Cell viability data are expressed as mean ± SE. Differences between the cell viabilities of control and treated groups were evaluated by one-way ANOVA followed by Dunnett’s t test. Probability value of p < 0.05 was considered statistically significant. Single asterisk (*) indicate p < 0.05; double asterisks (**) indicate p < 0.01; triple asterisks (***) indicate p < 0.001.

## Results

### The *n*-hexane extract of *T. camphoratus* exerts more potent effect than the ethanol extract on the growth inhibition of CL1-0 cells

**Fig. 1:**
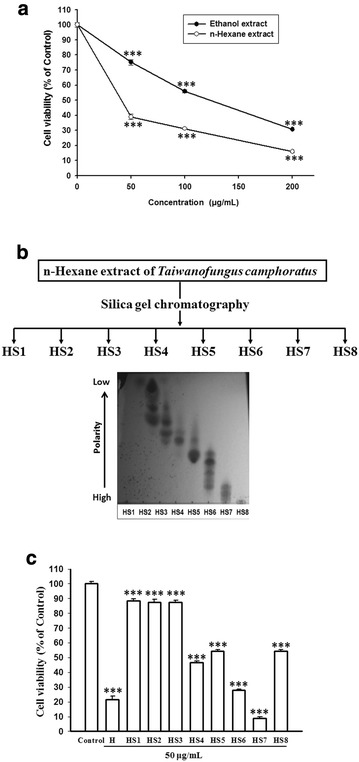
The activities of ethanol and *n*-hexane extracts of *T. camphoratus* and the eight separated fractions from n-hexane extract on the growth inhibition of CL1-0 lung cancer cells. **a** The cell viabilities of CL1-0 cells were measured after treatment with the ethanol or *n*-hexane extract of *T. camphoratus* for 72 h. **b** Eight fractions (HS1–HS8) were separated from the *n*-hexane extract by silica gel chromatography according to their polarities displayed in thin-layer chromatography. **c** The cell viabilities of CL1-0 cells were measured after treatment with *n*-hexane extract (H) or the separated fraction (HS1–HS8) at dose of 50 μg/mL as indicated for 72 h. Cell viability data (mean ± SE) are expressed as a percentage compared to the control and analyzed as described in “Statistical analysis”. ***p < 0.001 compared to the control group

To further investigate the anticancer activities of *T. camphoratus*, we extracted the *T. camphoratus* with ethanol and *n*-hexane, respectively, and compared their activities on the growth of CL1-0 cells. The result shows that both the two extracts inhibit the growth of CL1-0 cells in a dose-dependent manner and the *n*-hexane extract appears to have higher potency. As shown in Fig. 1a, the proliferation of CL1-0 cells was reduced to 30.7 and 31.2% of control by ethanol and *n*-hexane extracts at dose of 200 and 100 μg/mL, respectively. The *n*-hexane extract is at least two times more potent than the ethanol extract. We thus further separated the *n*-hexane extract to eight fractions (HS1–HS8) by silica gel chromatography according their polarity displayed in TLC (thin-layer chromatography) assay (Fig. 1b). These eight fractions were then examined for their activities on the growth inhibition of CL1-0 cells. The result shows that at dose of 50 μg/mL, the seventh fraction (HS7, the 7th fraction of silica gel chromatography after *n*-hexane extraction) exerts the highest potency (Fig. 1c). Similar results were also observed in PC3, HepG2, Hep3B and Huh7 cells (see Additional file 1). We thus chose the most potent HS7 for the subsequent experiments.

### Active fraction HS7 extracted from *T. camphoratus* profoundly inhibits the proliferation of CL1-0 cells

**Fig. 2:**
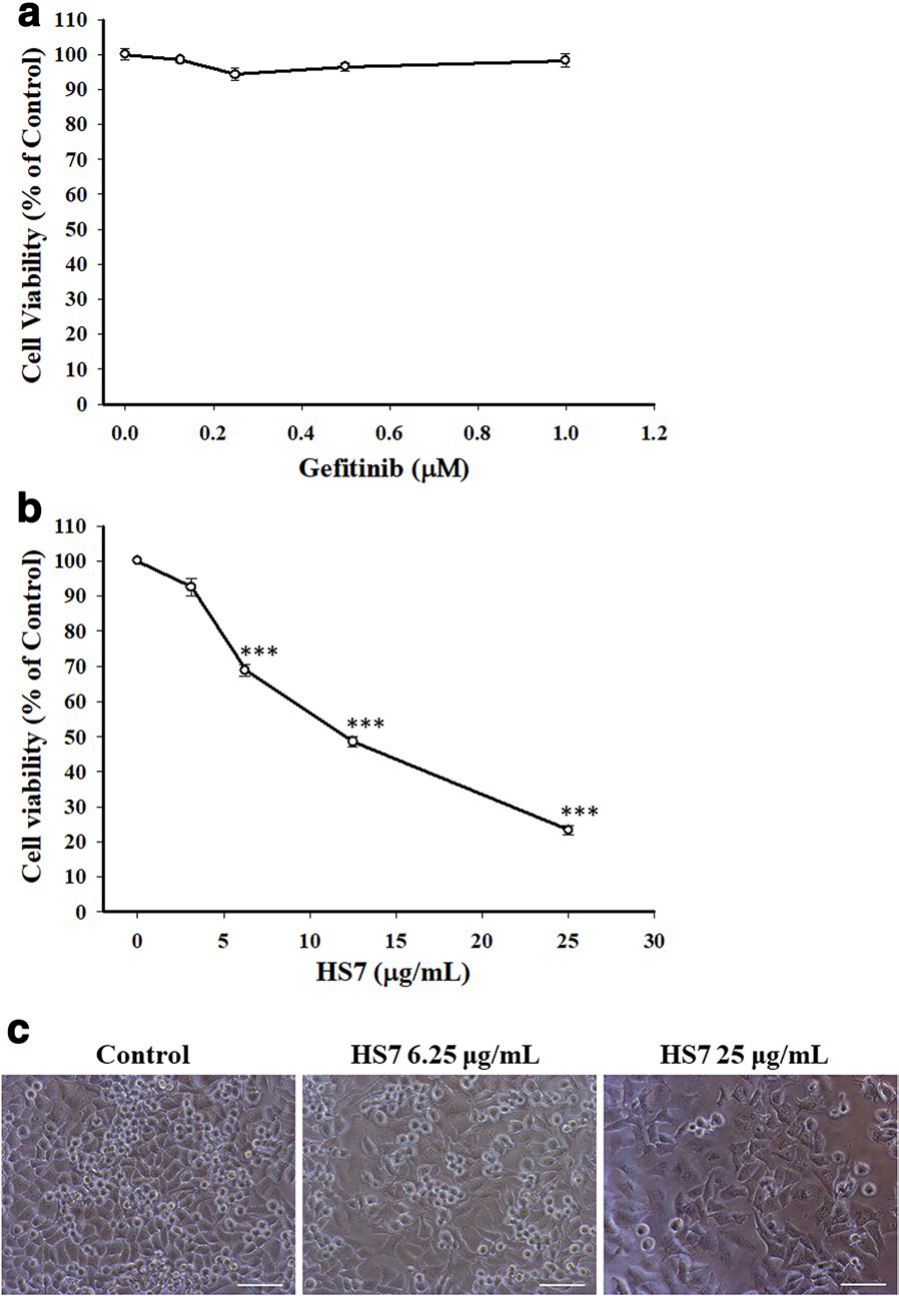
The effects of gefitinib (Iressa) and HS7 on the proliferation of CL1-0 lung cancer cells. **a** The cell viability of CL1-0 cells was measured after treatment with gefitinib for 72 h. **b** The cell viability of CL1-0 cells was measured after treatment with HS7 for 72 h. **c** The effect of HS7 on CL1-0 cells was examined by phase-contrast microscopy after 72 h of treatment, scale bar = 100 μm. Cell viability data (mean ± SE) are expressed as a percentage compared to the control and analyzed as described in “Statistical analysis”. ***p < 0.001 compared to the control group

**Fig. 3:**
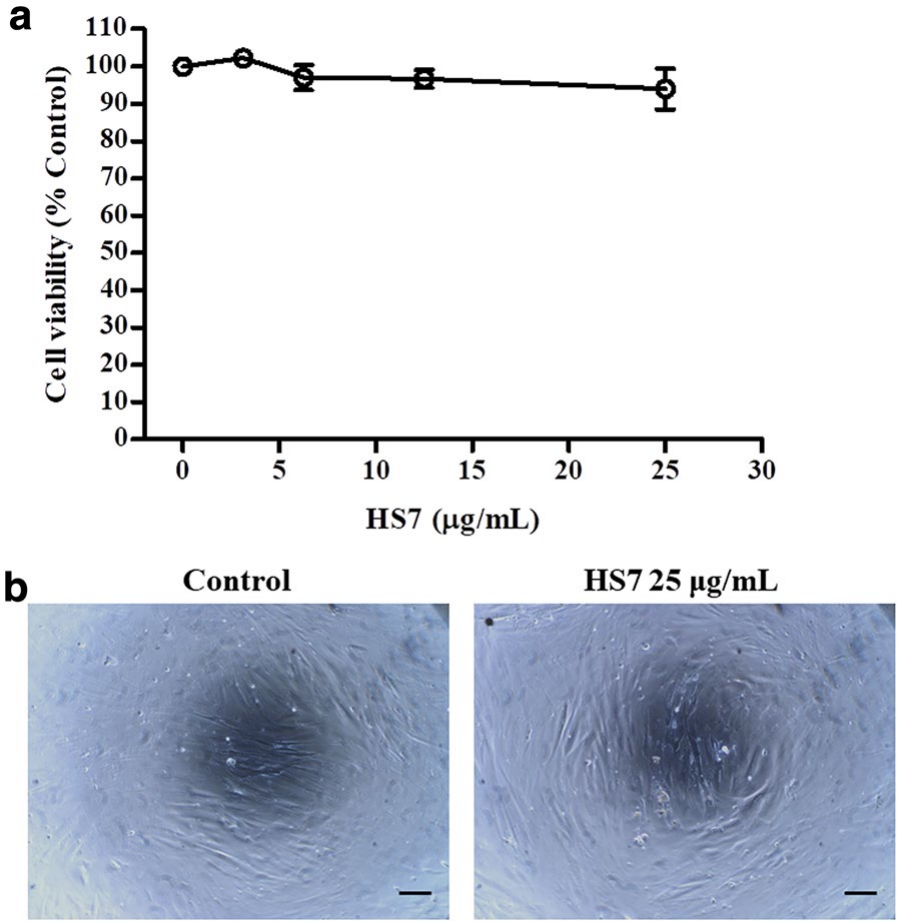
The effect of HS7 on the cell viability of MRC-5 normal fetal human lung fibroblasts. **a** The cell viability of MRC-5 cells was measured after treatment with HS7 for 72 h. **b** The effect of HS7 on MRC-5 cells was examined by phase-contrast microscopy after 72 h of treatment, scale bar = 100 μm. Cell viability data (mean ± SE) are expressed as a percentage compared to the control and analyzed as described in “Statistical analysis”

At first, we examined the effect of gefitinib (Iressa), a typical EGFR-TKI, on the proliferation of CL1-0 cells within its clinically achievable concentration (1 μM) [21]. As shown in Fig. 2a, consistent with the previous study by Lin et al. [19], gefitinib had no significant effect on the cell viability of EGFR wild-type CL1-0 cells. We then examined the effect of HS7 and found that it dose-dependently decreased the proliferation of CL1-0 cells after 72 h of treatment with IC50 of 11.4 μg/mL (Fig. 2b). Significant decrease of cell growth to 68.9% of control by HS7 could be found at dose of 6.25 μg/mL (Fig. 2b). When the dose was increased to 25 μg/mL, the proliferation of CL1-0 cells was suppressed to 23.4% of control (Fig. 2b). The inhibitory effect of HS7 was also confirmed by microscopic observation. As shown in Fig. 2c, significant decrease of CL1-0 cell number by HS7 could be observed at dose of 6.25 μg/mL and the effect was even more profound when the dose was increased to 25 μg/mL. By contrast, HS7 had no significant effect on the viability of MRC-5 cells, the normal fetal human lung fibroblasts, at doses within 25 μg/mL (Fig. 3a). The microscopic observation also showed the same result (Fig. 3b).

### HS7 induces apoptotic sub-G1 fraction in CL1-0 cells

**Fig. 4:**
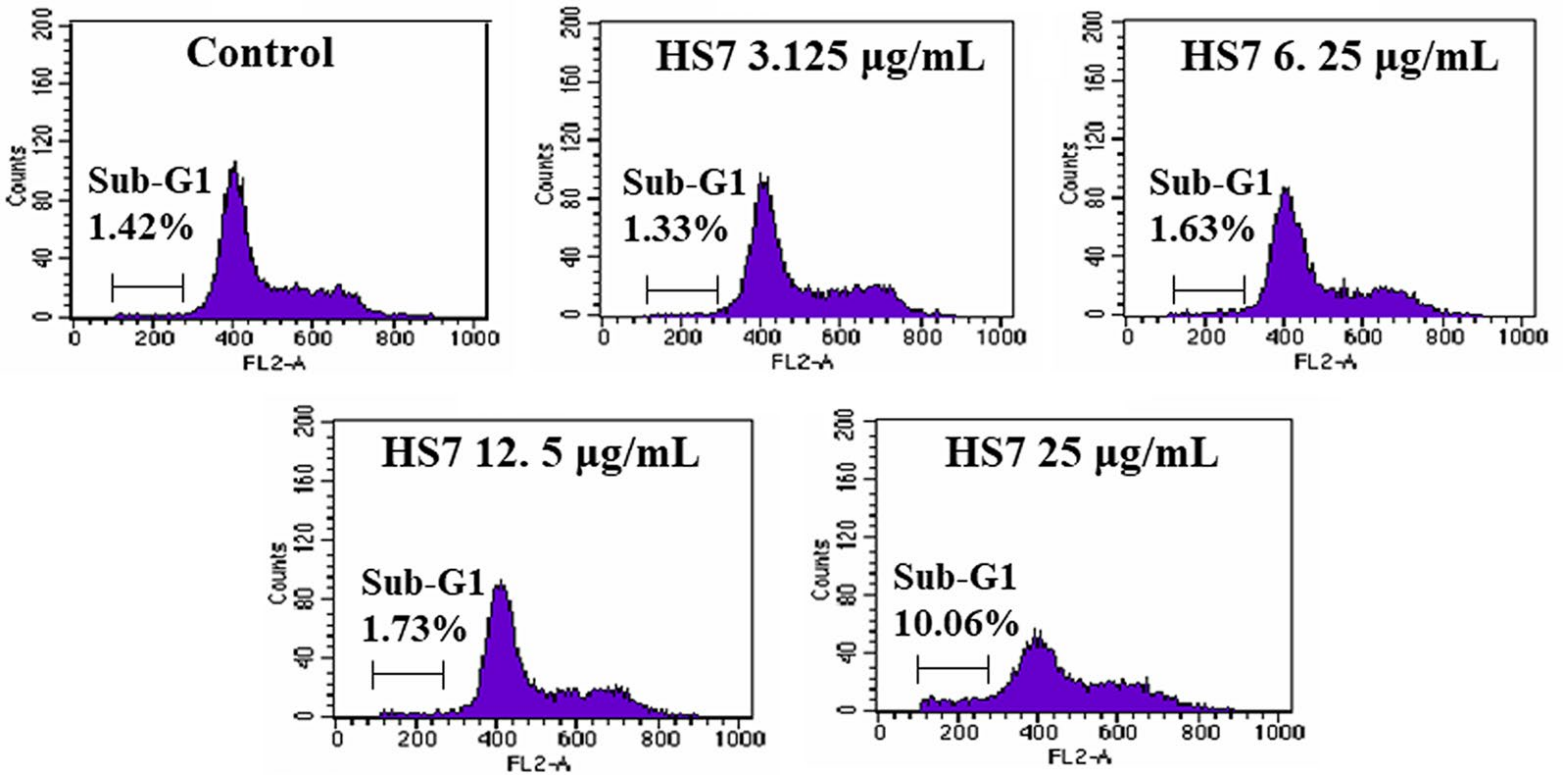
Representative flow cytometry histograms of CL1-0 lung cancer cells after treatment with HS7 for 72 h. HS7 increased the apoptotic sub-G1 fraction in CL1-0 cells at dose of 25 μg/mL but did not significant change the distribution of cells in the G0/G1, S and G2/M phases of the cell cycle within this dose. The percentage of cells in different phases of the cell cycle is shown in Table 1

After treatment with HS7 for 72 h, the CL1-0 cells were stained with PI and analyzed by flow cytometry to measure the change in cell-cycle distribution. As shown in Fig. 4 and Table 1, HS7 increased apoptotic sub-G1 fraction from 1.4% in control group to 10% at dose of 25 μg/mL but did not significantly change the distribution of cell populations in G0/G1, S and G2/M cell-cycle phases within this dose. Regarding the profound growth inhibition by HS7 shown in Fig. 2b, c, the progression of cell-cycle might be suppressed by HS7 through inhibition of the proliferative signaling pathways and the subsequent induction of CDK inhibitors that broadly inhibit each phase of the cell-cycle progression.

**Table 1:** Cell-cycle phase distribution (%) of 72 h HS7-treated CL1-0 cells

Treatment	Sub-G1 (%)^a^	Cell-cycle distribution		
		G0/G1 (%)	S (%)	G2/M (%)
Control	1.42	69.8	14.6	15.6
HS7 3.125 μg/mL	1.33	65.9	13.2	20.9
HS7 6.25 μg/mL	1.63	68.3	13.6	18.1
HS7 12.5 μg/mL	1.73	68	14.4	17.6
HS7 25 μg/mL	10.06	63.1	18.6	18.3

^a^Sub-G1 proportion is excluded from the percentage calculation of cell-cycle distribution

### HS7 inhibits AKT-mTOR signaling cascades in CL1-0 cells

**Fig. 5:**
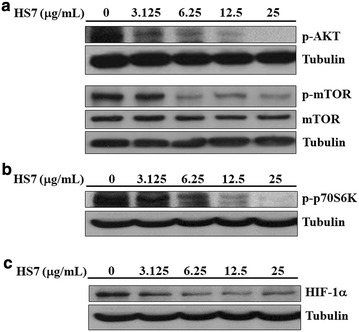
Inhibitory effects of HS7 on the AKT-mTOR signaling pathway and its downstream effectors in CL1-0 lung cancer cells after 72 h of treatment. **a** HS7 decreased the protein levels of phosphorylated AKT (p-AKT) and phosphorylated mTOR (p-mTOR) in a dose-dependent manner. **b** HS7 dose-dependently decreased the phosphorylated p70S6K protein (p-p70S6K). **c** HS7 dose-dependently decreased the HIF-1α protein. Cell lysates were analyzed by Western blot, using tubulin as loading control

The AKT-mTOR pathway is commonly dysregulated in NSCLC and plays a crucial role in tumourigenesis [4]. To investigate the molecular events underlying the inhibitory effect of HS7 on CL1-0 cell growth, we examined the AKT-mTOR signaling in HS7-treated cells. As shown in Fig. 5a, the constitutively activated/phosphorylated AKT protein (p-AKT) in CL1-0 cells was decreased in a dose-dependent manner after 72 h treatment with HS7. Marked decrease of activated/phosphorylated mTOR protein (p-mTOR) could be observed when the dose of HS7 was reached to 6.25 μg/mL (Fig. 5a).

The activated mTOR promotes protein translation and cell growth predominantly through activating p70S6K (ribosomal p70S6 kinase) [4, 22]. We thus examined the effect of HS7 on this downstream effector of AKT-mTOR signaling. In accordance with the result of Fig. 2b, HS7 significantly reduced the activated/phosphorylated p70S6K protein (p-p70S6K) in CL1-0 cells at the dose of 6.25 μg/mL and almost diminished the protein level when the dose was increased to 25 μg/mL (Fig. 5b).

In addition to p70S6K, the pivotal transcription factor HIF-1α (hypoxia-inducible transcription factor-1α) that regulates angiogenesis by inducing the expression of vascular endothelial growth factor (VEGF) is also a downstream target of mTOR [23, 24]. We next examined the change of HIF-1α in CL1-0 cells during mTOR inhibition by HS7. As shown in (Fig. 5c), the protein level of HIF-1α was also significantly downregulated when the dose of HS7 was reached to 6.25 μg/mL.

The suppression of these two important downstream effectors p70S6K and HIF-1α demonstrates the substantial inhibitory effects of HS7 on the AKT-mTOR signaling in CL1-0 cells.

### HS7 inhibits ERK and STAT3 signaling pathways in CL1-0 cells

**Fig. 6:**
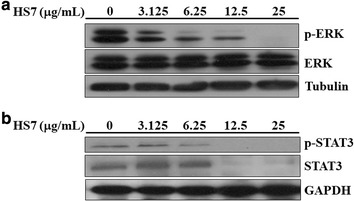
Inhibitory effects of HS7 on the ERK and STAT3 signaling pathways in CL1-0 lung cancer cells after 72 h of treatment. **a** HS7 decreased the protein levels of phosphorylated ERK (p-ERK) in a dose-dependent manner. **b** HS7 dose-dependently decreased both the protein levels of total (STAT3) and phosphorylated STAT3 (p-STAT3). Cell lysates were analyzed by Western blot, using tubulin or GAPDH as loading control

In addition to the AKT-mTOR pathway, the MEK-ERK pathway is also constitutively active in the majority of NSCLC cell lines [7] and patients [5]. It is considered to have a key role in the pathogenesis of lung cancer [25]. Intriguingly, HS7 also dose-dependently reduced the activated/phosphorylated ERK protein (p-ERK) in CL1-0 cells without significantly affecting the total ERK protein level (Fig. 6a).

The signal transducer and activator of transcription 3 (STAT3) is one of the three major downstream pathways (AKT-mTOR, ERK and STAT3) activated by EGFR phosphorylation, which promote proliferation and survival of NSCLC cells [9, 26]. Constitutive activation of STAT3 is also a common feature in NSCLC and it can be activated by JAK2 independent of known driver mutations [27]. We were interested to investigate if HS7 also inhibits this pro-survival and proliferative signaling in CL1-0 cells. As expected, HS7 significantly reduced the activated/phosphorylated STAT3 protein (p-STAT3) at dose of 6.25 μg/mL and almost diminished both the p-STAT3 and total STAT3 protein levels when the dose was increased to 12.5 μg/mL (Fig. 6b).

### HS7 is more effective than the synthetic inhibitors on the inhibition of signaling pathways in CL1-0 cells

**Fig. 7:**
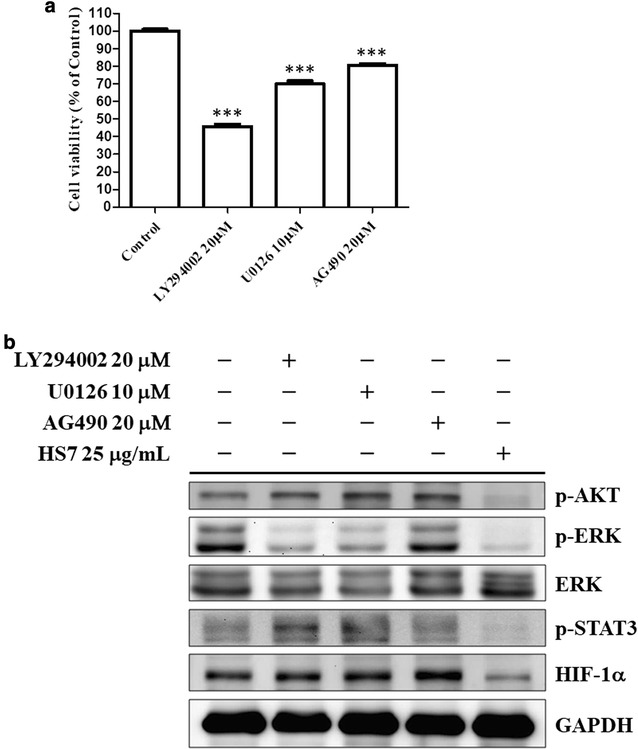
Effects of synthetic inhibitors and HS7 on the AKT ERK and STAT3 signaling pathways in CL1-0 lung cancer cells after 72 h of treatment. **a** The cell viabilities of CL1-0 cells were measured after treatment with LY294002 (PI3K-AKT inhibitor), U0126 (MEK-ERK inhibitor) or AG490 (JAK-STAT3 inhibitor) at indicated doses for 72 h. Cell viability data (mean ± SE) are expressed as a percentage compared to the control and analyzed as described in “Statistical analysis”. ***p < 0.001 compared to the control group. **b** The effects of LY294002, U0126, AG490 and HS7 on the protein levels of phosphorylated AKT (p-AKT), phosphorylated ERK (p-ERK), ERK, phosphorylated STAT3 (p-STAT3) and HIF-1α in CL1-0 cells. Cell lysates were analyzed by Western blot, using GAPDH as loading control

To compare the effects of HS7 with the synthetic inhibitors of these three signaling pathways, we treated CL1-0 cells with LY294002 (PI3K-AKT inhibitor), U0126 (MEK-ERK inhibitor), AG490 (JAK-STAT3 inhibitor) for 72 h at the doses which significantly reduced the proliferation of CL1-0 cells (Fig. 7a). At this 72 h treatment time point, LY294002 did not show inhibitory effect on the p-AKT protein but reduced the p-ERK protein as U0126 did (Fig. 7b). On the other hand, probably due to the feedback or compensatory activation at this time point, the reduction of p-ERK by U0126 or LY294002 was also accompanied with induction of p-AKT and p-STAT3 (Fig. 7b). The reduction of p-STAT3 by AG490 also was not observed at this time point, however, the compensatory induction of p-AKT was shown (Fig. 7b). Consistent with these feedback or compensatory inductions of p-AKT by these synthetic inhibitors, the protein levels of downstream component HIF-1α were also increased accordingly (Fig. 7b). In comparison, HS7 vigorously inhibits these three signaling pathways and HIF-1α without the feedback activation of pathway components or compensatory activation of parallel circuits induced by the synthetic inhibitors. This result implies that the multi-targeting HS7 might exert more effective anticancer activity than the single use of each synthetic signaling pathway inhibitor.

### HS7 induces CDK inhibitors in CL1-0 cells

**Fig. 8:**
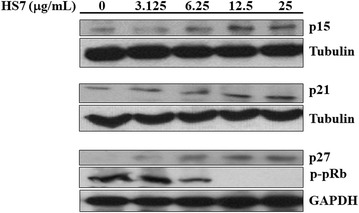
HS7 induces CDK inhibitors and inhibits phosphorylation of pRb protein in CL1-0 lung cancer cells after 72 h of treatment. HS7 dose-dependently increased protein levels of p15, p21 and p27, and decreased phosphorylated pRb protein (p-pRb). Cell lysates were analyzed by Western blot, using tubulin or GAPDH as loading control

**Fig. 9:**
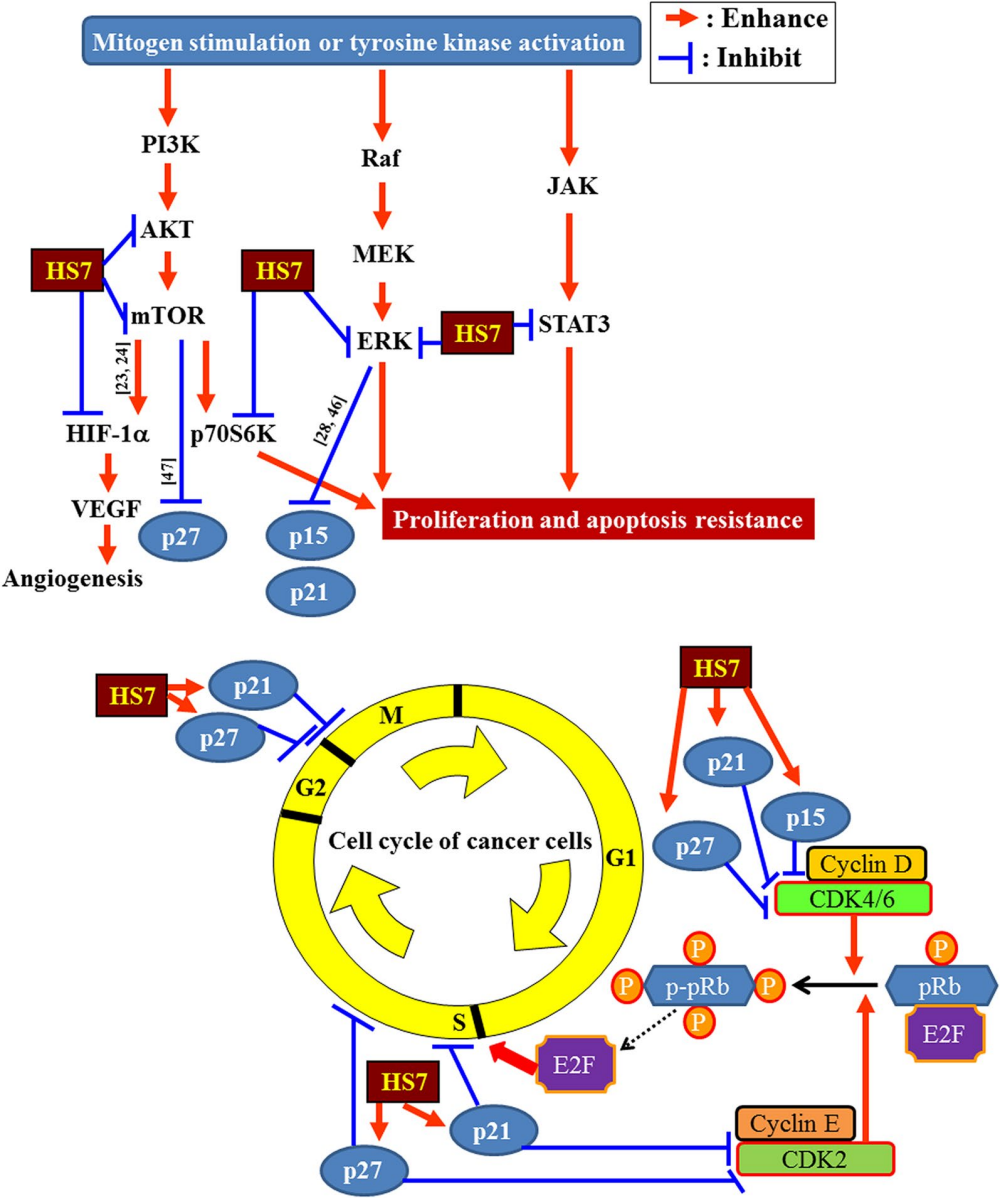
Schematic diagram displays the proposed mechanisms of action of HS7 in CL1-0 lung cancer cells. Through inhibition of AKT-mTOR, ERK and STAT3 signaling pathways, the active fraction HS7 from *T. camphoratus* increases the CDK inhibitors (p15, p21 and p27) and leads to cell cycle suppression and apoptosis induction. References for the proposed inhibition or enhancement are indicated along the arrow or T-bar. *ERK* extracellular signal-regulated kinases, *HIF-α* hypoxia-inducible factor 1-alpha, *JAK* Janus kinase, *MEK* mitogen-activated protein/extracellular signal-regulated kinase, *mTOR* mammalian target of rapamycin, *p70S6K* ribosomal p70 S6 kinase, *PI3K* phosphoinositide 3-kinase, *Raf* rapidly accelerated fibrosarcoma, *STAT3* signal transducer and activator of transcription 3, *VEGF* vascular endothelial growth factor

It has been shown that inhibition of AKT and ERK signaling by the typical EGFR-TKI gefitinib is accompanied with induction of CDK inhibitors such as p15, p21 and p27, and hypophosphorylation of retinoblastoma protein (pRb) [28, 29]. We thus examined the changes of these cell-cycle regulating components in HS7-treated cells. As expected, treatment with HS7 for 72 h dose-dependently increased the protein levels of p15, p21 and p27 (Fig. 8). As shown in the right-down side of Fig. 9, induction of these CDK inhibitors can prevent the pRb inactivation (phosphorylation) by CDK2/4/6 and the subsequent release of E2F to promote the G1/S transition of cell cycle [30]. Accordingly, the phosphorylation of pRb protein (p-pRb) was dose-dependently inhibited by HS7 while the CDK inhibitors (p15, p21 and p27) were induced (Fig. 8).

Significant induction of CDK inhibitors and inhibition of p-pRb were also observed when the dose of HS7 was reached to 6.25 μg/mL. This effective dose of HS7 is consistent with the aforementioned results shown in Figs. 2, 5 and 6, indicating the close correlation between the HS7-mediated events in CL1-0 cells. As proposed in Fig. 9, inhibition of these signaling pathways by HS7 might increase the CDK inhibitors (p15, p21 and p27) and lead to the suppression of cell cycle progression.

## Discussion

The oncogenic EGFR signaling in NSCLC engages the activation of downstream effectors such as AKT-mTOR, ERK and STAT3 to promote cell proliferation, cell survival, and tumor growth [4, 9, 31, 32]. Blockade of EGFR activation by TKIs targeted therapy has significantly changed the treatment paradigm in NSCLC [4]. However, only a small proportion of NSCLC patients can respond to clinically used TKIs [4, 31]. Most of the NSCLC patients do not carry the genetic alterations for the effectiveness of EGFR-TKIs treatment [13]. Tumor samples derived from NSCLC patients can show robust activation of AKT, ERK, and STAT3 while EGFR is not activated [31]. Alternative novel targeted therapeutics other than the EGFR-TKIs is imperatively needed. Nowadays, lots of ongoing efforts have been conducted in identifying potential therapeutics targeting the above-mentioned effectors (ERK, AKT-mTOR and STAT3), which are frequently dysregulated in NSCLC.

The anticancer activities of *T. camphoratus* (syn. *A. camphorata*) have been studied in numerous types of cancer cells including NSCLC [14, 16–18, 33, 34]. Its potential for the treatment of NSCLC has been shown in a preclinical evaluation in which the significant tumor suppression and apoptosis induction were observed [33]. To further develop the application of *T. camphoratus* for NSCLC treatment, we isolate a more potent active fraction (HS7) from its *n*-hexane extract. For the demanding of novel targeted therapeutics mentioned above, we investigate and demonstrate the substantial inhibition of ERK, AKT-mTOR and STAT3 signaling pathways by HS7 in EGFR-TKI resistant CL1-0 human NSCLC cells (EGFR wild-type).

The interplay between these pathways is complex in CL1-0 cells. Our results show that treatments with synthetic inhibitors of these pathways such as LY294002, U0126 and AG490 induce feedback or compensatory activation of parallel circuits in CL1-0 cells. This phenomenon is similar to that reported in other previous studies of synthetic inhibitors. The feedback activation of AKT plays an important role in the unsatisfactory clinical results of mTOR inhibitor in cancer treatment [35, 36]. Combining with AKT inhibitor to enhance the therapeutic effects of mTOR inhibitor for NSCLC treatment was thus suggested [35]. Our results show the simultaneous inhibition of p-AKT and mTOR signaling cascade (p-mTOR, p-p70S6K and HIF-1α) by HS7, implying its potential for this therapeutic strategy. On the other hand, targeting components of ERK signaling (RAS-RAF-MEK-ERK) also has been proposed for NSCLC treatment [37]. However, activation of alternative signaling pathways almost always occurs after inhibition of ERK [37]. Like that reported in the study by Hayashi et al. [38], inhibition of p-ERK by U0126 accompanied with increased p-AKT is also observed in our result. Combination with inhibitor of another parallel signaling tract such as AKT pathway has been studied in animal model [39] and clinical trial [37] for NSCLC treatment. Regarding the simultaneous suppression of both ERK and AKT-mTOR pathways by HS7, it might have the advantage over the individual use of synthetic ERK signaling inhibitors for NSCLC therapy.

Inhibition of STAT3 signaling may be effective for treatment of NSCLC irrespective of the EGFR mutation status [9]. In addition, activation of STAT3 also has been shown to participate in the resistance of NSCLC cells to erlotinib (EGFR-TKI) [9, 40] and radiation [9, 41]. An old FDA-approved anthelmintic drug niclosamide was recently found to overcome acquired erlotinib resistance and reverse radioresistance through suppression of STAT3 in NSCLC xenografts [40, 41]. In this perspective, the significant inhibition of STAT3 pathway by HS7 might also have the potential to reduce the resistance of NSCLC cells to EGFR-TKIs or radiotherapy. Further future investigation is warranted.

The success of targeted therapy can be limited by the ultimately developed resistance of cancer cells through mutation of the target kinase, signaling redundancy, feedback activation of pathway components, compensatory activation of parallel circuits and so forth [32]. One strategy to improve the efficacy is combination therapy [5, 22]. Therefore, simultaneously co-targeting the signaling pathways of such as AKT and ERK [5, 22, 39], mTOR and ERK [42], STAT3 and mTOR [43] have been proposed to improve the success of NSCLC targeted therapy. According to this strategy, the aforementioned multi-targeting activity of HS7 is worth of further development for its integrative use in NSCLC targeted therapy. The point of convergence targeted by HS7 to exert its multi-targeting effects is remained to be elucidated. It is also possible that the multi-components in HS7 are responsible for its multi-targeting activity. Compounds including polysaccharides, ergostan-type triterpenoids, a sesquiterpene, and phenyl and biphenyl derivatives had been isolated from *T. camphoratus* (syn. *A. camphorata*) [44, 45]. It is worthy to identify and quantify the active components in the HS7 fraction for the future development.

Like that observed in gefitinib (iressa)-treated cells [28, 29], inhibition of proliferative signaling cascades by HS7 is also accompanied with induction of p15, p21 and p27, and decrease of pRb phosphorylation. It is reported that suppression of proliferative signaling like ERK and AKT-mTOR could inhibit the degradation of p21 and p27, respectively [46, 47], and up-regulate the p15 mRNA level [28]. HS7 might also modulate the proteolysis or transcription of these CDK inhibitors by the similar ways, resulting in inhibition of pRb phosphorylation. On the other hand, in addition to constrain the cell-cycle progression, HS7 also induced apoptosis at dose of 25 μg/mL, and massive apoptosis induction (46% sub-G1 fraction) could be observed at higher dose up to 50 μg/mL (see Additional file 3). The profound inhibition of these targeted signaling effectors by HS7 did have apoptotic killing effect on CL1-0 cells.

## Conclusions

From *T. camphoratus*, we isolate an active fraction (HS7) that simultaneously inhibits the signaling pathways (AKT-mTOR, ERK and STAT3) and induces CDK inhibitors (p15, p21 and p27) in EGFR TKI resistant CL1-0 NSCLC cells, resulting in growth inhibition and apoptosis induction (Fig. 9). Combinations of synthetic signaling pathway inhibitors for NSCLC targeted therapy have been proposed by many studies; however, none of them are clinically available at present. This multi-targeting activity of HS7 implies its potential as an alternative medicine for the treatment of EGFR TKIs resistant NSCLC.

## Additional files

**Additional file 1.** The activities of eight separated fractions (HS1-HS8) from the *n*-hexane extract of *Taiwanofungus camphoratus* on the growth inhibition of four cancer cell lines.

**Additional file 2.** Minimum standards of reporting checklist-R.

**Additional file 3.** Apoptosis induction in CL1-0 lung cancer cells by HS7 at dose of 50 μg/mL.

## Abbreviations

CDK: cyclin-dependent kinase
ERK: extracellular signal-regulated kinase
EGFR: epidermal growth factor receptor
GAPDH: glyceraldehyde-3-phosphate dehydrogenase
HIF-1α: hypoxia-inducible factor 1α
JAK: Janus kinase
MEK: mitogen-activated protein/extracellular signal-regulated kinase
mTOR: mammalian target of rapamycin
p70S6K: ribosomal p70 S6 kinase
PBS: phosphate-buffered saline
PI3K: phosphoinositide 3-kinase
pRb: retinoblastoma protein
Raf: rapidly accelerated fibrosarcoma
SRB: sulforhodamine B
STAT3: signal transducer and activator of transcription 3
TKI: tyrosine kinase inhibitor
VEGF: vascular endothelial growth factor
