# Supplementary material for: Active fraction (HS7) from Taiwanofungus camphoratus inhibits AKT-mTOR, ERK and STAT3 pathways and induces CDK inhibitors in CL1-0 human lung cancer cells
Source: Chin Med. 2017 Nov 15;12:33. doi: 10.1186/s13020-017-0154-9 (PMC5688709; doi:10.1186/s13020-017-0154-9)
Supplement: Supplementary file 1 — Additional file 1. The activities of eight separated fractions (HS1-HS8) from the n-hexane extract of Taiwanofungus camphoratus on the growth inhibition of four cancer cell lines. [file 13020_2017_154_MOESM1_ESM.docx]

**
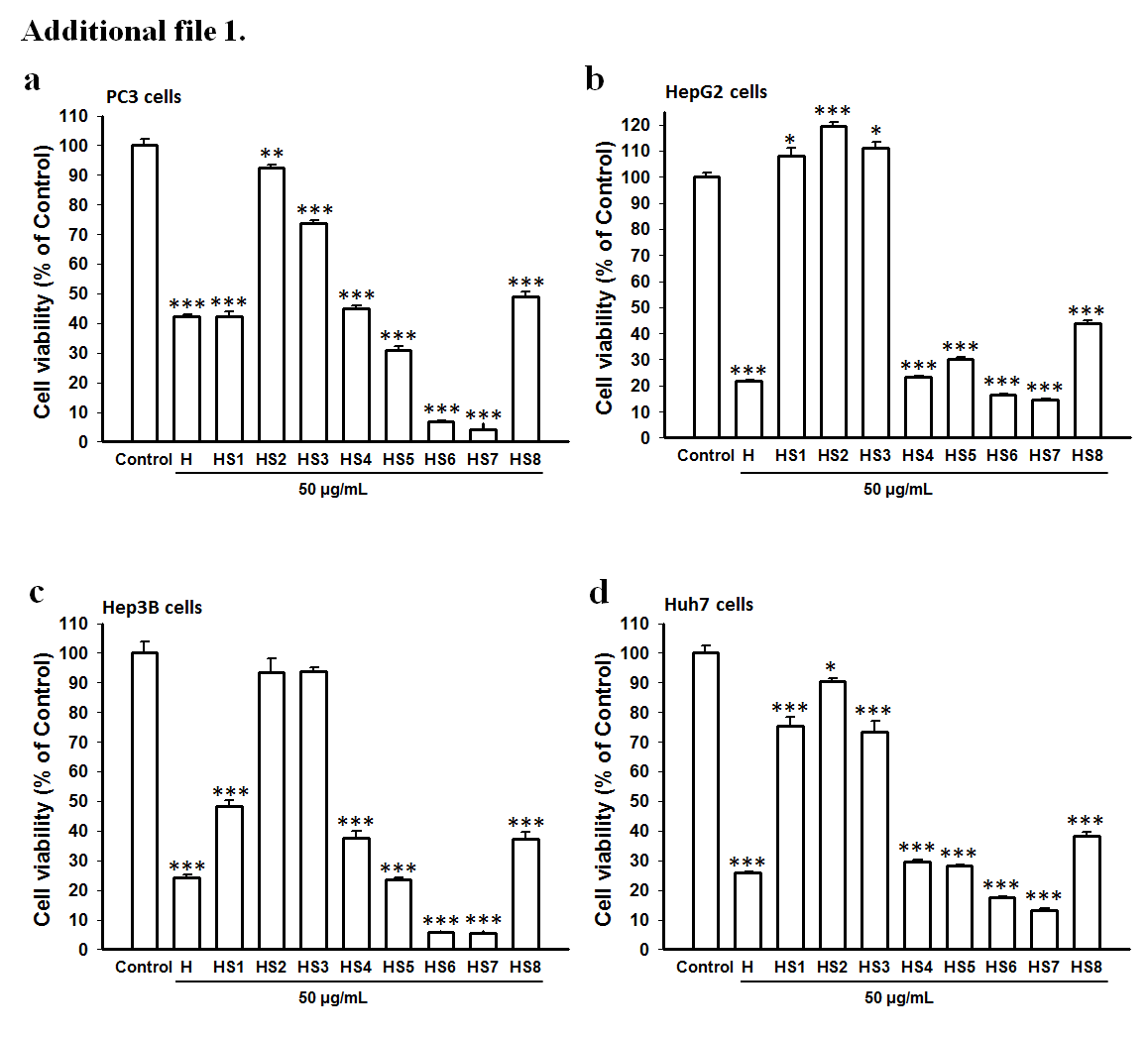
Additional file 1.** The activities of eight separated fractions (HS1-HS8) from the n-hexane extract of *Taiwanofungus camphoratus* on the growth inhibition of four cancer cell lines. **a** PC3 prostate cancer, **b** HepG2, **c** Hep3B and **d** Huh7 hepatocellular carcinoma cells were treated with the n-hexane extract (**H**) or the separated fraction (**HS1** to **HS8**) at dose of 50 μg/mL for 72 h and then the cell viabilities were measured. Cell viability data (mean ± SE) are expressed as a percentage compared to the control and analyzed as described in Statistical Analysis. Single asterisk (*) indicate p<0.05; double asterisks (**) indicate p<0.01; triple asterisks (***) indicate p<0.001. The cells were maintained in DMEM medium supplemented with 10% FBS and cultured at 37°C in a water-jacketed 5% CO_2_ incubator. The seeding densities of cells in 96-well plate (cells/well) were 3000 (PC3, Hep3B), 5000 (Huh7) and 6000 (HepG2), respectively.
