# Supplementary material for: Active fraction (HS7) from Taiwanofungus camphoratus inhibits AKT-mTOR, ERK and STAT3 pathways and induces CDK inhibitors in CL1-0 human lung cancer cells
Source: Chin Med. 2017 Nov 15;12:33. doi: 10.1186/s13020-017-0154-9 (PMC5688709; doi:10.1186/s13020-017-0154-9)
Supplement: Supplementary file 2 — Additional file 2. Minimum standards of reporting checklist-R. [file 13020_2017_154_MOESM2_ESM.docx]

Minimum Standards of Reporting Checklist

*BioMed Central* advocates full and transparent reporting. Please ensure that your paper provides the information requested below where applicable. On submitting your paper you will be asked to confirm you have included this information, or give reasons for any instances where it is not made available. You will also be asked to upload this file and it should be cited in the Methods section.

# Experimental design and statistics

The following information should be included in the Methods section and inserted in the table below:

| **Question** | **Answer** |
| --- | --- |
| 1. The exact sample size (n) for each experimental group/condition (as a number, not a range). Include details of a power analysis if done, or any other relevant considerations that determined the choice of sample size. For n < 6, individual data values should be shown rather than summary statistics alone. | One-way ANOVA, followed by Dunnett's t-test was utilized to analyze the cell viability data shown in Figs. 1, 2, 3, 7 and additional files 1. Data were expressed as mean ± standard error (SE), where n=6. |
| 2. A description of sample collection that enables the reader to understand whether the samples represent technical or biological replicates, and an explanation of inclusion/exclusion criteria if samples or organisms were excluded from the analysis. | The fruiting body-like *Taiwanofungus camphoratus* (voucher number TC-2004-09-001) was cultivated and provided by Well Shine Biotechnology Development Co. (Taipei, Taiwan). The air-dried ground powder of cultivated *Taiwanofungus camphoratus* was extracted exhaustively with n-hexane or ethanol. The n-hexane extract was then further separated to eight fractions (HS1 to HS8) by silica gel chromatography according to their polarities displayed in thin-layer chromatography (Fig. 1b).  The CL1-0 human lung adenocarcinoma cell line was kindly provided by Dr. Shine-Gwo Shiah (NHRI, Miaoli, Taiwan). The MRC-5 normal fetal human lung fibroblasts and cell lines (PC3, HepG2, Hep3B and Huh7) used in the cell viability test shown in additional file 1 were obtained from the Bioresource Collection and Research Center (BCRC, Taiwan). |
| 3. How samples/ organisms were allocated to experimental groups and processed, and full details of the randomisation procedure used (if relevant). | To compare the anticancer activities of ethanol or n-hexane extract of *Taiwanofungus camphoratus*, we treated cancer cells (cell densities were described in the manuscript) with these extracts at the same dose range, respectively, and examined their effects in the cell viability by SRB assay as described in Methods.  To compare the anticancer activities of the eight fractions (HS1-HS8) form n-hexane extract of *Taiwanofungus camphoratus*, we treated cancer cells (cell densities were described in the manuscript) with these fractions at the dose of 50 μg/mL, and examined their effects in the cell viability by SRB assay as described in Methods.  To investigate the anticancer activities of the most potent fraction HS7, we treated cancer cells (cell densities were described in the manuscript) with HS7 at the dose rage of 3.125-25 μg/mL, and examined its effects in the cell viability, cell-cycle, and various functional protein levels as described in Methods. |
| 4. For sample assessment by human investigators, a statement on whether the investigator was blinded to group assignment and outcome assessment, and how this blinding was achieved and evaluated (if relevant). | Not applicable |

| 5. How many times each experiment shown was replicated and an indication of the extent of variation from experiment to experiment. | Most of the experiments were performed two or three times. |
| --- | --- |
| 6. Information on the statistical methods and measures used. It should be clear whether the tests are one-sided or two-sided, whether there are adjustments for multiple comparisons, whether medians or means are being shown, whether error bars are standard deviations (SD), standard error of mean (SEM) or confidence intervals. | One-way ANOVA, followed by Dunnett's t-test was utilized to analyze the cell viability data shown in Figs. 1, 2, 3, 7 and additional files 1. Data were expressed as mean ± standard error (SE), where n=6. |
| 7. A justification for the appropriateness of statistical tests used to assess significance. Do the data meet the assumptions of the tests? Is there an estimate of variation within each group of data, and is the variance similar between groups that are being statistically compared?  In addition, information essential to interpreting the data presented should be made available in the figure and table legends. If the study involves health interventions for human participants, please refer to the relevant reporting guidelines from the EQUATOR Network, and the Biosharing Portal for reporting checklists for biological and biomedical research, where applicable. | In the cell viability data (Figs. 1, 2, 3, 7 and additional files 1), each drug-treated group was compared to the respective vehicle-treated control group. Differences between control and treated groups were evaluated by one-way ANOVA followed by Dunnett’s t-test. Probability value of p<0.05 was considered statistically significant. Single asterisk (*) indicate p<0.05; double asterisks (**) indicate p<0.01; triple asterisks (***) indicate p<0.001. |

# Research involving humans

If your research involved humans, please confirm you have adhered to the relevant reporting guideline from the [EQUATOR Network](http://www.equator-network.org/), and included the completed checklist as an additional file with your submission:

|  | **Answer** (page and line number inserted/Not applicable for my study) |
| --- | --- |
| - I have followed the relevant reporting for my study type, and included a populated checklist with my submission - Not applicable for my study | Not applicable for my study. |

# Resources

A description of all resources used should be included in the Methods section, with enough information to allow them to be uniquely identified. The table below should be completed with confirmation that this was done (i.e. included in the Methods section) or is not applicable. If this has not been completed, but is applicable, you should contact the journal editorial staff before proceeding.

|  | **Answer** (page and line number inserted/Not applicable for my study) |
| --- | --- |
| •Antibodies: report source, catalogue code, characteristics, dilutions and how they were validated for the system under study. | The information is shown in the Method section (page 11, line number 229-235). |
| •Cell lines: report source, whether identity has been authenticated and whether tested for mycoplasma contamination. We encourage researchers to check the NCBI database for contamination of cell lines. | The CL1-0 human lung adenocarcinoma cell line was kindly provided by Dr. Shine-Gwo Shiah (NHRI, Miaoli, Taiwan). The MRC-5 normal fetal human lung fibroblasts and cell lines (PC3, HepG2, Hep3B and Huh7) used in the cell viability test shown in additional file 1 were obtained from the Bioresource Collection and Research Center (BCRC, Taiwan).  The information of CL1-0 and MRC-5 cells is shown in the Method section (page 7, line number 156-162). In the legend of additional file 1, the seeding densities of cells in 96-well plate (cells/well) were 3000 (PC3, Hep3B), 5000 (Huh7) and 6000 (HepG2), respectively. These four cell lines were maintained in DMEM medium supplemented with 10% FBS and cultured at 37°C in a water-jacketed 5% CO2 incubator. |
| •Organisms: report source, species, strain, sex, age, husbandry, inbred and strain characteristics of transgenic and mutant animals. | It is not applicable for my study. |
| •Tools (software, databases and services): report standard tool name, provider and version number, if available. For antibodies, model organisms (mice, zebrafish and flies) and tools, authors are strongly encouraged to cite Research Resource Identifiers (RRIDs). To do so, please go to the Resource Identification Portal to search for your research resource and insert the reference text into your Methods section. | Page 10, line number 210-211.  The percentages of cell-cycle distribution were calculated by CellQuest software (BD Bioscience, CA, USA). |

# Availability of data and materials

The table below should be completed with confirmation that this was done (i.e. included in the Methods section) or is not applicable.

|  | **Answer** (page and line number inserted/Not applicable for my study) |
| --- | --- |
| All datasets on which the conclusions of the paper rely must be either deposited in publicly available repositories (where available and ethically appropriate) or presented in the main paper or additional supporting files, in machine-readable format whenever possible. If authors are unable to fulfil this requirement, they should contact journal editorial staff, after checking our list of Recommended Repositories. | We attached the supplementary data in additional files 1 and 2. |
| Links to deposited datasets, or datasets in additional files, should be explicitly referenced in a section entitled “Availability of Data and Materials”. Guidance on where to deposit your data can be found on the Availability of Data and Materials policy page. | It is not applicable for my study. |
| If computer code was used to generate results that are central to the paper’s conclusions, include a statement in the “Availability of data and materials” section to indicate how the code can be accessed. Include version information and any restrictions on availability. For deposited data and published code, a full reference with an accession number, doi or other unique identifier should be included in the reference list. | It is not applicable for my study. |
| If reproducible materials are generated as a result of the research (for example new animal mutants), a statement on their availability should be included. | Page 7-8, line number 166-171.  The fruiting body-like *Taiwanofungus camphoratus* (voucher number TC-2004-09-001) was cultivated and provided by Well Shine Biotechnology Development Co. (Taipei, Taiwan). The air-dried ground powder of cultivated *Taiwanofungus camphoratus* was extracted exhaustively with n-hexane or ethanol. The n-hexane extract was then further separated to eight fractions (HS1 to HS8) by silica gel chromatography according to their polarities displayed in thin-layer chromatography (Fig. 1b). |
