# Supplementary material for: Active fraction (HS7) from Taiwanofungus camphoratus inhibits AKT-mTOR, ERK and STAT3 pathways and induces CDK inhibitors in CL1-0 human lung cancer cells
Source: Chin Med. 2017 Nov 15;12:33. doi: 10.1186/s13020-017-0154-9 (PMC5688709; doi:10.1186/s13020-017-0154-9)
Supplement: Supplementary file 3 — Additional file 3. Apoptosis induction in CL1-0 lung cancer cells by HS7 at dose of 50 μg/mL. [file 13020_2017_154_MOESM3_ESM.docx]

**
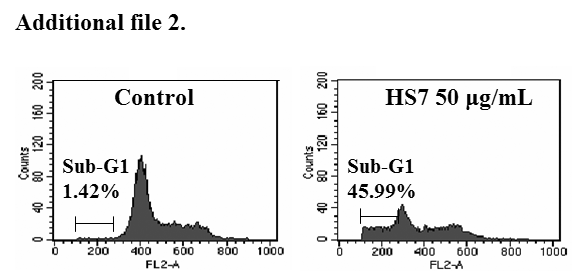
**

**Additional file 2.** Apoptosis induction in CL1-0 lung cancer cells by HS7 at dose of 50 μg/mL. The sub-G1 apoptotic fraction in CL1-0 cells was analyzed by flow cytometry after treatment with 50 μg/mL of HS7 for 72 h.
